# Concurrent and lagged effects of psychosocial job stressors on symptoms of burnout

**DOI:** 10.1007/s00420-019-01437-0

**Published:** 2019-05-20

**Authors:** Emina Hadžibajramović, Gunnar Ahlborg, Anna Grimby-Ekman

**Affiliations:** 1 Institute of Stress Medicine, Region Västra Götaland, Carl Skottsbergs gata 22 B, SE-413 19 Gothenburg, Sweden; 2 Health Metrics, Department of Public Health and Community Medicine, Institute of Medicine, Sahlgrenska Academy, University of Gothenburg, Gothenburg, Sweden; 3 Occupational and Environmental Medicine, Department of Public Health and Community Medicine, Sahlgrenska Academy, University of Gothenburg, Gothenburg, Sweden

**Keywords:** Psychosocial work environment, Longitudinal analysis, Criterion-based approach, Job demands, Decision authority, Effort–reward imbalance, Interaction

## Abstract

**Purpose:**

Burnout is a mental condition described as being a result of long-term stressors commonly related to psychosocial factors at work. The aim of the present study was to investigate longitudinal relationships between job demands, decision authority, effort and reward, and symptoms of burnout, as well as the joint effects of job demands and decision authority, and of effort and reward.

**Methods:**

The data came from a four-wave longitudinal cohort study of Swedish health care workers. Longitudinal associations were analysed using mixed effects regression models with random intercept.

**Results:**

The concurrent analysis showed that demand and decision authority, as well as effort and reward, were associated with symptoms of burnout over time. Evidence of the lagged effects of workplace factors on burnout symptoms was limited to reward. No clear effect modification was found.

**Conclusion:**

An increase in unfavourable working conditions implied increasing scores on the burnout measure over time. The concurrent effects of job demands, decision authority, effort and reward on symptoms of burnout were seen. The evidence of lagged effects was limited to the low-reward condition. Regularly monitoring these work environment conditions at workplaces can help identify risk situations for burnout and thus be useful in the prevention of work-related mental illness. Lastly, a new approach to defining the risk groups was proposed, which is consistent across different populations and time points.

**Electronic supplementary material:**

The online version of this article (10.1007/s00420-019-01437-0) contains supplementary material, which is available to authorized users.

## Introduction

Burnout is a mental condition originally described as the result of long-term stressors related to psychosocial conditions at work (Maslach 1976; Melamed et al. 1992). Later, the concept was broadened to include also stressors in private life. The burden of mental and somatic symptoms due to burnout is high, leads to long-term sick leave and has a high public health impact (Glise et al. 2012; Seidler et al. 2014). Burnout is generally measured by the self-report instruments and the theoretical basis for the available instruments differs. The Maslach Burnout Inventory (MBI) is one of the most often used instruments, where burnout is defined as a psychological multidimensional construct including emotional exhaustion, cynicism or depersonalization and reduced personal accomplishment (Maslach and Jackson 1981; Maslach et al. 2001). The Shirom–Melamed Burnout Questionnaire (SMBQ) is another instrument, where emotional exhaustion, physical fatigue and cognitive weariness represent the core component of burnout (Melamed et al. 1992, 2006; Shirom 2003). By this approach, burnout represents a separate construct not interchangeable with depression, although they share some common variance (Shirom 2003).

Several reviews have described associations between psychosocial risk factors and various mental health outcomes such as stress-related disorders (Nieuwenhuijsen et al. 2010), depressive disorders or clinical depression (Madsen et al. 2017; Netterstrom et al. 2008; Rugulies et al. 2017; Theorell et al. 2015) and common mental disorders (Harvey et al. 2017; Stansfeld and Candy 2006). As regards the psychosocial risk factors, the most frequently used theoretical stress models are the job demand-control (JDC) model and the effort–reward imbalance (ERI) model. The most unfavourable working conditions are characterised by the joint effects of the two dimensions, i.e. the combination of high demands and low control (job strain), and the combination of high effort and low reward (effort–reward imbalance) (Karasek 1979; Karasek and Theorell 1990; Siegrist 1996).

Although accumulated evidence points to a relationship between unfavourable working conditions and mental health problems such as depression and anxiety, there is still limited evidence regarding burnout (Aronsson et al. 2017). A review examining the association between psychosocial working conditions and burnout and its core component emotional exhaustion only identified six methodologically adequate longitudinal studies (Seidler et al. 2014). The most recent review including 25 studies, had a broader approach regarding exposure factors and outcome, including besides emotional exhaustion also cynicism and reduced personal accomplishment among employees (Aronsson et al. 2017). They concluded that high demands, low job control, high work load, low reward and job insecurity increased the risk of emotional exhaustion, while high levels of job support and workplace justice hade a protective effect. In 18 out of 25 studies, the MBI or versions of MBI were used as outcome measures and the SMBQ in only one study. The scientific evidence of the effect of job strain on burnout was judged insufficient since it was investigated in only one high-quality study (Aronsson et al. 2017). In that study, the association between job strain and burnout was found (Ahola and Hakanen 2007). None of the studies in the abovementioned review investigated the effects of effort–reward imbalance.

Moreover, certain limitations are highlighted in many abovementioned reviews. For instance, the evidence presented for many factors is based on few studies for each factor (Nieuwenhuijsen et al. 2010). Lack of methodologically adequate, high-quality longitudinal studies is a further limitation (Aronsson et al. 2017; Harvey et al. 2017), as is the need to conduct longitudinal studies with multiple time intervals, i.e. more than two waves (Tang 2014). Such a design would help to analyze whether associations between psychosocial risk factors and symptoms of burnout are concurrent or if lagged effects are present, e.g. after more than 1 year. Adding knowledge about temporal relationships would be helpful from a preventive perspective. Another issue concerns the classification and analysis of psychosocial factors based on ordinal data, which has been a topic of discussion for decades (Kampen and Swyngedouw 2000). Both JDC and ERI as well as many other are multi-item questionnaires are based on ordinal responses but are often used as continuous measures. Uncritical and unreflective use of ordinal data as linear is associated with a certain degree of risk (Grimby et al. 2012). Although these scales tend to be linear in the middle of the scale, it may not be the case towards the ends of the scale, which in turn compromises important properties of responsiveness and sensitivity (DeVellis 2006; Hadžibajramović 2015). Moreover, groups at risk for adverse health effects are often found towards the ends of scales. The common approach for the JDC is to define risk groups based on the population median split and for the ERI imbalance on the effort–reward ratio but there are other approaches as well (Fransson et al. 2012; van Vegchel et al. 2005). Comparability between studies and different approaches for risk groups of JDC have been discussed (Choi et al. 2015) and as regards the ERI, concerns have been raised regarding questionnaire with a two-step procedure (Tsutsumi et al. 2008).

Abovementioned approaches for JDC and ERI are dependent on empirical data distribution which makes it difficult to compare results between different studies, besides being not optimal for ordinal data (Stevens 1955; Tennant and Conaghan 2007). There are other approaches which provide solutions to these problems, e.g. using a criterion-based classification (CBA) of the psychosocial factors (Hadžibajramović 2015). The CBA scores are defined by experts in the particular field of interest on the basis of theoretical knowledge. The CBA scores are based on the frequency distribution of the item responses into predefined response combinations, i.e. independent of empirical data distribution and in addition are easy to interpret.

Thus, the aim of this study was to investigate whether there is a relationship between job demands, decision authority, effort and reward and symptoms of burnout, more specifically, to examine whether possible associations between psychosocial exposures and outcome are delayed and/or occur simultaneously. In addition to investigating the individual effect of each workplace factor using an alternative method, we also wanted to evaluate whether there are any joint effects of job demand and decision authority, on the one hand, and of effort and reward, on the other hand, on symptoms of burnout.

## Methods

### Study design and population

The data come from a four-wave longitudinal cohort study of employees in a large public healthcare organisation, Region Västra Götaland in Sweden. Baseline data were collected by means of a postal questionnaire sent to a random sample of 5300 out of 48,000 employees in 2004 (T1), with follow-ups in 2006 (T2), 2008 (T3) and 2010 (T4). An inclusion criterion of at least 1 year of employment (at least 50% of a full-time position) was applied. Two reminders were sent to non-responders. Written informed consent for participation in the study was obtained from the participants. The study was approved by the Regional Ethical Review Board in Gothenburg, Sweden, and it was conducted in accordance with the 1964 Declaration of Helsinki.

The response rate at T1 was 61% (*n* = 3209) and at follow-up of those eligible (still employed and participation in a previous wave) at T2 was 83% (*n* = 2665), T3 83% (*n* = 1970) and T4 72% (*n* = 1422). The mean age at baseline was 46.7 years and 86% were women. The three most common professions were nurse (46%), assistant nurse (16%) and physician (7%). Further demographic and study-specific details are available in published studies (Glise et al. 2010; Lundgren-Nilsson et al. 2012). Burnout was measured at all four waves (T1–T4), and JDC and ERI at the first three (T1–T3).

### Measures

#### Psychosocial factors

The use of a sum score, which is the most common approach for the calculation of total scores for the two dimensions of the DCQ and of ERI (explained below), should not be taken for granted when calculated on ordinal data (Stevens 1955; Tennant and Conaghan 2007). Consequently, the classification in this study into high, medium and low levels of demand and decision authority and of effort and reward was done using the criterion-based approach (CBA) (Hadžibajramović 2015).

JDC was measured using the Demand-Control Questionnaire (DCQ) (Theorell et al. 1988). In this study, all the demand items and the two decision authority items, a sub-dimension of the control, were used. All the items were expressed as questions with four frequency-based response options (often, sometimes, seldom, never). The classification into low, medium and high levels of demands and decision authority was done using the criterion-based approach (CBA) described in detail in Appendix 1.

The effort dimension of the Effort–Reward Imbalance (ERI) Questionnaire consists of six items. One item regarding physical load is usually excluded when evaluating white-collar workers, which was also the case in the present study. The reward dimension was operationalised using 11 items, divided into esteem (five items), promotion (four items) and job security (two items). All items were formulated as statements and responded to in a two-step procedure. First, subjects agree or disagree on an item statement. Then if they agree, subjects are asked to evaluate on a four-point Likert scale the perceived distress connected to this (not at all distressed/somewhat distressed/distressed/very distressed). The total scores for each dimension were defined by the CBA and described in Appendix 1.

#### Outcome measure

The Shirom–Melamed Burnout Questionnaire (SMBQ) was used to measure symptoms of burnout (Melamed et al. 1992). The SMBQ originally contained 22 items with four subscales: physical fatigue (eight items), cognitive weariness (six items), tension (four items) and listlessness (four items). In the present study, a revised 18-item version (tension excluded) was used and proved to have good construct validity (Lundgren-Nilsson et al. 2012). All items are expressed as statements and are rated using a seven-point response scale (almost never to almost always). Instead of the mean score of the 18 items, a recommended transformed score was calculated (Lundgren-Nilsson et al. 2012). This score ranges from 18 to 126, with higher values indicating a high degree of burnout, and a value of 79 cut-off for clinical burnout.

### Statistical analysis

Descriptive statistics were given in percentages for categorical variables, and means and standard deviation (SD) for continuous variables. Longitudinal associations analysed using mixed effects regression models with random intercept regression coefficients along with the 95% confidence interval (CI) were presented as measures of association. Associations between each workplace factor (job demands, decision authority, effort, reward) and burnout were evaluated from two different time aspects as defined in equations below. In the concurrent effects models, both exposures and the outcome were measured simultaneously over three waves (T1–T3). In the lagged effect models, exposures were measured at time *t* − 1 (T1–T3), i.e. 2 years before the outcome, and outcome at time *t* (T2–T4):

$$Y_{it} = \beta_{0i} + \sum\limits_{j = 1}^{J} {\beta_{1j} X_{itj} + \beta_{2} t + \varepsilon_{it} } \left( {\text{concurrent effects model}} \right),$$

$$Y_{it} = \beta_{0i} + \sum\limits_{j = 1}^{J} {\beta_{1j} X_{it - 1j} + \beta_{2} t + \varepsilon_{it} } \left( {\text{lagged effects model}} \right),$$

where *Y*_*it*_ are observations for subject *i* at time *t*, *β*_0*i*_ is the random intercept, *X*_*ijt*_ is the independent variable *j* for subject *i* at time *t*, and *β*_1*j*_ is the regression coefficient for independent variable *j*, *J* is the number of independent variables, *β*_2_ is the regression coefficient for indicator of time *t* in the first equation and *t* − 1 in the second, and *ε*_*it*_ is the “error” for subject *i* at time *t*.

Burnout measured at time *t* − 1 (SMBQ-lag) was evaluated as a possible confounder, recognising that it could influence both workplace factors and SMBQ at time *t*. In both models, the cross-sectional and the longitudinal relationships are “pooled” together into a single regression coefficient. In other words, the regression coefficients combine the “between-subjects” (i.e. relationship between absolute values at each time point) and the “within-subject” (i.e. relationships between changes between subsequent time points). Note that adding the SMBQ-lag in the models removes the cross-sectional part from the analyses (Twisk 2003).

Also evaluated as possible confounders were physical activity during the preceding 3 months [(1) mostly sedentary, (2) light, at least 2 h per week or, (3) more intensive exercise at least 2 h per week or high intensity several times per week]; length of education [(1) short = assistant nurse, caretaker, dental nurse, secretary, cleaning staff and administrative assistant, (2) long = nurse, physician, dentist, physiotherapist, ward manager, dietician, speech therapist, psychologist, almoner, biomedical scientist, technician and engineer]; social support outside the work (no/yes), gender and age.

Regression models were fitted separately for each workplace factor following the purposeful model building strategy (Bursac et al. 2008). In the first step, time and SMBQ-lag was evaluated in the model along with the workplace factor (model 1). In the second step, time and possible confounders were added to the model if they fulfilled the recommended confounder criteria (model 2) (Rothman et al. 2008). In the third step, all workplace factors were tested in the same model along with the confounders.

Joint exposure effects of job demands and decision authority, and of effort and reward were investigated by creating combined variables of the two factors, this to examine effect modification. Although there were nine possible response combinations given that each factor consisted of three categories, some combinations were judged to be fairly similar in terms of the amount of exposure. Consequently, the combined variables each consisted of four categories. Taking demand/decision authority as an example, the four categories were (1) joint exposure (high/low), (2) single-exposure demand (high/medium or high/high), (3) single-exposure decision latitude (medium/low or low/low) and (4) low or medium exposure (low/high, medium/high, low/medium or medium/medium). Effect modification of the joint effects was analysed as a departure from additivity, i.e. checking whether the joint-exposure effect was different from the sum of two single-exposure effects. Departure from additivity can be evaluated by examining whether the interaction contrast (IC) defined as below is significantly different from zero (Rothman et al. 2008):

$${\text{IC}} = {\text{Risk}}_{{{\text{joint}}\;{ \exp }}} + {\text{Risk}}_{{{\text{single}}\;{ \exp }1}} + {\text{Risk}}_{{{\text{single}}\;{ \exp }2}} - {\text{Risk}}_{{{\text{no}}\;{ \exp }}} .$$

In the context of linear regression, we calculated the IC along with accompanied 95% confidence interval, by plugging in the estimated means from the regression models in the equation above.

Two sensitivity analyses were carried out to test the robustness of the results and to investigate for possible healthy worker effect. First, the participants in the study were compared with the drop-outs at each follow-up (T2, T3 and T4) with regard to their baseline values for the workplace factors, SMBQ, gender and age. In the second sensitivity analysis, we analysed a subsample consisting of individuals who participated at all three occasions and compared to the results from the main analysis.

## Results

### Descriptive statistics

Frequency distributions of the workplace factors across different time points are shown in Table 1. Mean burnout symptoms at different time points were (SD): 70.35 (11.81), 69.16 (11.92), 68.21 (12.02) and 68.37 (12.48) for T1, T2, T3 and T4, respectively. The mean of the individual standard deviations over time for SMBQ was 5.39.

**Table 1:** Descriptive statistics showing the proportion (%) and count (*n*) of workplace factors at baseline (T1), and at the first (T2) and second (T3) follow-up

	T1% (*n*)	T2% (*n*)	T3% (*n*)
Demand^a^			
Low	16 (513)	18 (465)	19 (366)
Medium	64 (2000)	65 (1692)	66 (1268)
High	20 (628)	17 (455)	15 (291)
Decision^a^			
Low	39 (1245)	39 (1034)	42 (805)
Medium	27 (851)	27 (706)	28 (542)
High	34 (1088)	34 (885)	31 (590)
Effort^b^			
Low	20 (617)	22 (553)	27 (499)
Medium	52 (1606)	53 (1345)	52 (971)
High	28 (862)	26 (663)	21 (399)
Reward^b^			
Low	5 (154)	3 (73)	5 (89)
Medium	14 (410)	10 (237)	12 (206)
High	81 (2417)	87 (2052)	83 (1450)

^a^Demand-Control Questionnaire, decision authority = subscale of the control dimension

^b^Effort–Reward Questionnaire

### Concurrent effects

All workplace factors were associated with symptoms of burnout (*p* values < 0.0001) in both models, Table 2 (complete regression models in Appendix 2). From the results in Table 2, model 1, high demands increased the burnout score by 6.02 points compared to low demands. The corresponding figure for medium demands was 2.93, indicating a dose–response relationship. The same patterns were observed for all other workplace factors. Although attenuated, the associations remained after adjusting for confounders (SMBQ-lag, physical activity, social support and age) (model 2) and after adjusting workplace factors for each other (Table 3). Gender and education did not qualify as confounders. Time was statistically significant in all models in the first step but in not in the second or third step.

**Table 2:** Longitudinal analysis showing concurrent effects between burnout symptoms measured using SMBQ and each of the workplace factors: regression coefficients (Coeff) and 95% confidence interval (CI) (complete models are shown in Appendix)

	Model 1^e^		Model 2^f^	
	Coeff.	95% CI	Coeff.	95% CI
Demand^a^				
High	6.02	5.24; 6.80	3.50	2.63; 4.39
Medium	2.93	2.34; 3.52	1.76	1.08; 2.44
Low	0	0	0	
Decision^a^				
Low	3.58	2.98; 4.17	1.99	1.38; 2.60
Medium	2.11	1.56; 2.67	1.15	0.48; 1.82
High	0	0	0	
Effort^b^				
High	5.67	5.00; 6.35	3.61	2.85; 4.36
Medium	2.30	1.76; 2.85	1.33	0.70; 1.97
Low	0	0	0	
Reward^b^				
Low	6.11	5.02; 7.20	4.70	3.28; 6.13
Medium	3.18	2.51; 3.86	2.71	1.83; 3.60
High	0	0	0	
JDC^c^				
Joint exposure	5.37	4.55; 6.19	3.67	2.69; 4.65
Demand only	3.12	2.36; 3.88	1.56	0.58; 2.53
Decision only	2.03	1.49; 2.56	1.20	0.62; 1.78
No exposure	0		0	
ERI^d^				
Joint exposure	7.87	6.32; 9.41	5.82	3.79; 7.86
Effort only	3.93	3.37; 4.50	2.83	2.15; 3.52
Reward only	5.62	4.16; 7.07	4.37	2.40; 6.35
No exposure	0		0	

Shirom–Melamed Burnout Questionnaire

^a^Demand-Control Questionnaire, decision authority = subscale of the control dimension

^b^Effort–Reward Questionnaire

^c^Job demand-control

^d^Effort–reward imbalance

^e^Adjusted for time

^f^Adjusted for social support, physical activity, age and SMBQlag

**Table 3:** Longitudinal analysis showing concurrent effects between burnout symptoms measured using SMBQ and the workplace factors: regression coefficients (Coeff) and 95% confidence interval (CI)

	Coeff.	95% CI	*p* value
Demand^a^			0.048
High	1.22	0.13; 2.31	
Medium	0.91	0.13; 1.69	
Low	0		
Decision^a^			< 0.0001
Low	1.53	0.88; 2.18	
Medium	0.90	0.19; 1.61	
High	0		
Effort^b^			< 0.0001
High	2.95	2.02; 3.88	
Medium	0.81	0.08; 1.53	
Low	0		
Reward^b^			< 0.0001
Low	3.77	2.32; 5.22	
Medium	2.32	1.41; 3.23	
High	0		
Social support			< 0.0001
No	2.51	1.35; 3.67	
Yes	0		
Physical activity			< 0.0001
Sedentary	4.54	3.63; 5.44	
Light	1.28	0.69; 1.88	
Moderate/intense		0	
SMBQlag	0.64	0.61; 0.66	< 0.0001
Intercept	19.96	18.25; 21.66	< 0.0001

Shirom–Melamed Burnout Questionnaire

^a^Demand-Control Questionnaire, decision authority = subscale of the control dimension

^b^Effort–Reward Questionnaire

Regarding the joint effects of demands and decision authority, respectively, effort and reward, departure from additivity was not statistically significant as the IC and its accompanied 95% CI were 0.91 (− 0.49; 2.31) and 1.38 (− 1.48; 4.25) for the DCQ and the ERI, respectively.

### Lagged effects analysis

Lagged effects models are shown in Table 4 and Appendix 2. In the first step, all workplace factors had a *p* value < 0.0001, except decision authority where *p* = 0.059 (model 1). Dose–response relationships were seen for all factors, meaning that the effect on outcome increased with increasing level of exposure compared to no exposure. After adjusting for confounders (SMBQ-lag, physical activity and social support) reward was the only workplace factor that remained statistically significant (model 2). The same was observed when all workplace factors were included in the same model (results not shown). Consequently, joint effects were not investigated. Time was significant in the first step models but not in the second or third step.

**Table 4:** Longitudinal analysis showing lagged effects between burnout symptoms measured using SMBQ and each of the workplace factors: regression coefficients (Coeff) and 95% confidence interval (CI) (complete models are shown in Appendix)

	Model 1^e^		Model 2^f^	
	Coeff.	95% CI	Coeff.	95% CI
Demand^a^				
High	2.84	1.94; 3.74	0.18	− 0.60; 0.96
Medium	1.07	0.40; 1.75	0.11	− 0.51; 0.73
Low	0			
Decision^a^				
Low	0.82	0.14; 1.51	0.21	− 0.33; 0.75
Medium	0.55	− 0.10; 1.10	0.01	− 0.60; 0.58
High	0			
Effort^b^				
High	1.82	1.03; 2.61	0.31	− 0.37; 1.00
Medium	0.70	0.05; 1.34	0.07	− 0.52; 0.66
Low	0			
Reward^b^				
Low	3.54	2.28; 4.80	1.18	0.02; 2.34
Medium	2.46	1.69; 3.22	1.46	0.72; 2.20
High	0	0		
JDC^c^				
Joint exposure	2.51	1.56; 3.47	0.69	− 0.16; 1.54
Demand only	1.57	0.70; 2.44	− 0.49	− 1.31; 0.33
Decision only	0.41	− 0.20; 1.03	0.01	− 0.51; 0.53
No exposure	0			
ERI^d^				
Joint exposure	3.88	2.08; 5.69	1.37	− 0.33; 3.06
Effort only	1.22	0.58; 1.88	0.39	− 0.20; 0.98
Reward only	2.86	1.17; 4.54	0.74	− 0.83; 2.32
No exposure	0		0	

Shirom–Melamed Burnout Questionnaire

^a^Demand-Control Questionnaire, decision authority = subscale of the control dimension

^b^Effort–Reward Questionnaire

^c^Job demand-control

^d^Effort–reward imbalance

^e^Adjusted for time

^f^Adjusted for social support, physical activity, age and SMBQlag

### Sensitivity analyses

A significant difference in SMBQ baseline values was found between participants and dropouts at T2, the mean values being 70.01 and 72.06, respectively (*p* < 0.0001), and between participant and dropouts at T3, with mean values of 70.01 and 71.5 (*p* < 0.0001), respectively. No significant differences were found when workplace factors were considered. The proportion of men among dropouts at T2 (17%) and T3 (15%) was higher compared to the participants at T1 (13%) (*p* values 0.026 and 0.023, respectively). There was also a significant difference in age. The proportion of participants aged 55 years or older was 26%, compared to 29, 35 and 34% among the dropouts at T2, T3 and T4, respectively.

Additional analysis was done on the subsample consisted of individuals who participated at all three occasions, resulting in the same final models and negligible changes in coefficient estimates (data not shown).

## Discussion

Two time aspects were examined. In the concurrent analysis, all workplace factors were associated with burnout symptoms. This is in line with previous research on other mental health outcomes (Harvey et al. 2017; Madsen et al. 2013; Rugulies et al. 2017; Theorell et al. 2015). In the lagged analysis, only reward was significantly associated with burnout symptoms. To our knowledge, this is the first time that the ERI has been used in a longitudinal study with more than two waves and with burnout measured using the SMBQ, and thus also the first time that both models have been evaluated simultaneously. Previous studies have shown that the JDC and the ERI models have complementary effects on mental distress (Calnan et al. 2004; Theorell 2017). This is now also confirmed for the symptoms of burnout. According to the theoretical models, the joint effects are more harmful than the sum of individual effects of each factor. In this study, no clear results of effect modification due to joint effects were observed.

Lagged effects of the reward should be confirmed in other studies before a more reliable conclusion about this can be reached. The usefulness of a time-lag model depends on the biological plausibility. It is important that time lag suits the purpose of the study and the aetiology of the relationships between the investigated variables. A possible explanation for why no other associations were found could be that the lag of 2 years is too long and that concurrent effects are more plausible. Concurrent effects imply that stressors are associated with simultaneous levels of burnout. This explanation is also in accordance with a previous review, where it was suggested that stressors and experiences of stress on the one hand and burnout symptoms on the other, change simultaneously (Shirom et al. 2006). Furthermore, it can be argued that the burnout scores at one time point influence both the perception of workplace conditions and burnout score at a later time point. To control for reversed causality, the SMBQ-lag was included as a confounder. However, its impact on the burnout scores was not very strong. Besides shedding light on the temporal relationships, this study also adds to deepening the knowledge of the burnout phenomenon using the SMBQ as a burnout measure, which is conceptually different from the most used burnout measure, the MBI.

To our knowledge, formally test of the job strain and the ERI effects were not previously done with burnout as outcome. In many studies, instead of separate factors, job strain or ERI is included as predicting variables of some health-related outcome, but the formal test of whether departure from additivity is present or not is not performed. In a study by Ahola et al., job strain is included as a predictor for burnout measured by the MBI (Ahola and Hakanen 2007). In the present study, we offer an easy method of how the interaction can be tested in the context of linear regression.

The study is based on a relatively large sample size, which can imply good power and more reliable estimations, and on four waves, which enables a longitudinal analysis approach. A methodological strength is the handling of ordinal data according to the modern measurement theories. We propose, for example, the CBA approach as a new way of defining the risk groups of demand, decision authority, effort and reward. How exposure groups are defined and the comparability of different measures of exposures is a subject of discussion (Choi et al. 2015). There are several approaches to defining the risk groups in the JDC and the ERI (Fransson et al. 2012; van Vegchel et al. 2005). The advantage of the CBA is that the scores are not based on the sum values and better suit the ordinal data. A further strength is that the risk groups were defined in collaboration with experts in the field and are easily described in words. As opposed to the median split, the CBA scores are independent of empirical data distribution, making them consistent over time and comparable between different populations and studies.

One limitation is that the results are only valid for similar populations of human service organisations, and the generalisability to other populations is limited. Another limitation was that all measurements were based on self-reported questionnaire data, dependent on the participants’ recall and comprehension of the survey items. On the other hand, all the instruments were well established and validated according to modern psychometric theory. In this study, only the decision authority items are used and not the complete control dimension. The sub-dimension skill discretion was considered difficult to interpret in the context of this study since demands related to skills and learning are nowadays inherent to in highly professional work such as healthcare and are, therefore, expected.

Indications of healthy worker effects were found when comparing participants’ with non-participants’ burnout baseline, which could be a source of bias. However, based on additional analysis on subsample of those participating on all occasions, we cannot conclude that this is the case in our sample. No differences between the two groups were found regarding the workplace factors.

Lastly, a practical implication of importance is that the results of this study indicate the possibility of early discovery of people at risk of developing a clinical burnout condition. The clinical relevance of the SMBQ and its usability as a screening tool was confirmed previously (Lundgren-Nilsson et al. 2012). As the burden of mental and somatic symptoms due to burnout is often high and long lasting (Glise et al. 2012), preventing and minimising the onset of burnout at an early stage is of crucial public health interest. A practical implication from this study is that it is important to frequently monitor all the abovementioned workplace factors and attempt to minimise the experiences of unfavourable working conditions whenever possible.

## Conclusion

Unfavourable psychosocial working conditions are risk factors for the development of burnout symptoms over time. The concurrent effects of job demands, decision authority, effort and reward on symptoms of burnout were seen. The evidence of lagged effects was limited to the low-reward condition. Practical implication of the study is that regularly monitoring workplace conditions in a workplace investigation is recommended to identify early signs of future burnout cases and to prevent the high burden of mental and somatic symptoms due to burnout. Lastly, a new approach for defining the risk groups was proposed, which is consistent across different populations and time points.

## Electronic supplementary material

Below is the link to the electronic supplementary material.

Supplementary material 1 (PDF 498 kb)

Supplementary material 2 (PDF 479 kb)
