# Supplementary material for: Concurrent and lagged effects of psychosocial job stressors on symptoms of burnout
Source: Int Arch Occup Environ Health. 2019 May 20;92(7):1013–21. doi: 10.1007/s00420-019-01437-0 (PMC6768907; doi:10.1007/s00420-019-01437-0)
Supplement: Supplementary file 1 — Supplementary material 1 (PDF 498 kb) [file 420_2019_1437_MOESM1_ESM.pdf]

Appendix 1 - Criterion based approach

The use of a sum score, which is the most common approach for the calculation of total scores for the two dimensions of the DCQ and of ERI, should not be taken for granted when calculated on ordinal data,[1, 2]. Consequently, the classification in this study into high, medium and low levels of demand and decision authority and of effort and reward was done using the criterion-based approach (CBA),[3]. The CBA scores are defined by experts in the particular field of interest on the basis of theoretical knowledge. The scores are based on the frequency distribution of the item responses into predefined response combinations.

In this study, the classification process were conducted in three steps. First, a group of experts in statistics, occupational and environmental medicine and stress research within the research group worked out a classification scheme and a proposed classification of each scale dimension. This was sent to three external experts, including Professor Töres Theorell regarding demand and decision authority, Professor Johannes Siegrist and PhD Göran Fáhlen regarding effort and reward, who, in the second step, were requested to scrutinize the proposal and suggest re-classification wherever they disagreed with the proposed classification. Finally, the research group adjusted the classification in line with the few changes suggested by the external experts, which resulted in a final version.

The CBA scores of demand and decision authority were calculated as follows. The response alternatives for each item were first classified as low, medium or high responses for demands and decision authority, respectively. As regards the demand items and the two decision authority items, *never* and *seldom* were considered to be low responses, and *often* a high response. The direction of the responses for one demand item was reversed (*Do you have sufficient time for all your work tasks?*). For that item *often* was considered a low response and *never* or *seldom* high responses. The response alternative *sometimes* was considered to be a medium response for all the items. Finally, a CBA score (low, medium, high) was calculated for each dimension based on the frequency distribution of the items into predefined categories, shown in Table 1 and 2. A high level of demands was defined as at least three out of five items with high responses. A low level of demands was defined as at least three items with low responses and none with high responses. All other response combinations were considered to be medium level. A low level of decision authority was defined as being at least one out of the two items with low responses and none with high responses, and vice versa for the high level of decision authority. All other response combination were considered to be medium level of decision authority.

The CBA scores for the ERI were defined as follows. The response alternatives *distressed* and *very distressed* were considered to be high effort and low reward responses, and s*omewhat distressed* was considered to be a medium response. If answering *yes* in the first step led to the evaluation of distress, then response alternatives *no* and *yes, but not at all distressed* were considered to be low effort and high reward responses. If *no* required evaluation then *yes* and *no, but not at all* distressed were considered to be high reward responses. CBA scores of effort were shown in Table 3 and defined as follows: low level of effort = three or more items rated with a low response and no item with a high response; high effort = three or more high responses and no low response. All other response combinations were considered to be medium level. The three sub-dimensions of reward were combined into a total reward score. At least two high levels out of the three sub-dimensions were considered to be high level of reward and vice versa for the low levels. The high levels of esteem were achieved by having three or more high responses for esteem items and no more than one low response. Three or more low responses corresponded to low esteem. At least two high responses and a maximum of one low response resulted in high promotion levels, and at least two low responses resulted in low promotion levels. At least one high response and no low response defined high security levels. The reversed was true for low security levels.

REFERENCES

1. Stevens, S.S., On the averaging of data. *Science*1955;121(3135):113-6.
2. Tennant, A. and P.G. Conaghan, The Rasch measurement model in rheumatology: What is it and why use it? When should it be applied, and what should one look for in a Rasch paper? *Arthritis Care Res.*2007;57(8):1358-62.
3. Hadžibajramović, E., *Aspects of validity in stress research*, in *Institute of Medicine at Sahlgrenska Academy*. 2015, University of Gothenburg.

Table 1. The low, medium and high levels of decision authority according to the criterion approach (CBA) based on the number of responses found in the response categories often, sometimes, never and seldom, out of two decision authority items in the Demand-Control Questionnaire.

Response profiles	Never or seldom	Sometimes	Often	CBA score
[1]	2	0	0	Low
[2]	1	1	0	Low
[3]	1	0	1	Medium
[4]	0	2	0	Medium
[5]	0	1	1	High
[6]	0	0	2	High

Table 2. The low, medium and high levels of demands according to the criterion approach (CBA) based on the number of responses found in the response categories often, sometimes, never and seldom, out of five demand items in the Demand-Control Questionnaire.

Response profiles	Often	Sometimes	Never or seldom	CBA score
[1]	5	0	0	High
[2]	4	1	0	High
[3]	4	0	1	High
[4]	3	2	0	High
[5]	3	1	1	High
[6]	3	0	2	High
[7]	2	3	0	Medium
[8]	2	2	1	Medium
[9]	2	1	2	Medium
[10]	2	0	3	Medium
[11]	1	4	0	Medium
[12]	1	3	1	Medium
[13]	1	2	2	Medium
[14]	1	1	3	Medium
[15]	1	0	4	Medium
[16]	0	5	0	Medium
[17]	0	4	1	Medium
[18]	0	3	2	Medium
[19]	0	2	3	Low
[20]	0	1	4	Low
[21]	0	0	5	Low

Table 3. The low, medium and high levels of effort according to the criterion approach (CBA) based on the number of responses found in the response categories low, medium and high out of five effort items in the Effort-Reward Questionnaire. Low effort responses = No or Yes, but not at all distressed, medium effort response =somewhat distressed high effort responses = distressed or very distressed.

Response profiles	Low effort responses	Medium response	High effort responses	CBA score
[1]	5	0	0	Low
[2]	4	1	0	Low
[3]	4	0	1	Low
[4]	3	2	0	Low
[5]	3	1	1	Low
[6]	3	0	2	Medium
[7]	2	3	0	Medium
[8]	2	2	1	Medium
[9]	2	1	2	Medium
[10]	2	0	3	Medium
[11]	1	4	0	Medium
[12]	1	3	1	Medium
[13]	1	2	2	Medium
[14]	1	1	3	High
[15]	1	0	4	High
[16]	0	5	0	Medium
[17]	0	4	1	Medium
[18]	0	3	2	Medium
[19]	0	2	3	High
[20]	0	1	4	High
[21]	0	0	5	High
