# Supplementary material for: Concurrent and lagged effects of psychosocial job stressors on symptoms of burnout
Source: Int Arch Occup Environ Health. 2019 May 20;92(7):1013–21. doi: 10.1007/s00420-019-01437-0 (PMC6768907; doi:10.1007/s00420-019-01437-0)
Supplement: Supplementary file 2 — Supplementary material 2 (PDF 479 kb) [file 420_2019_1437_MOESM2_ESM.pdf]

APPENDIX 2

Table 2 Longitudinal analysis showing concurrent effects between burnout symptoms measured using SMBQ[1] and each of the workplace factors; regression coefficients (Coeff), 95% confidence interval (CI). All p-values (type III) were <0.0001 in both models.

	Model 1*			Model 2**		
	Coeff.	*95% CI*	*p-value*	*Coeff.*	*95% CI*	*p-value*
Intercept	67.31	66.67;67.95	<0.0001	20.56	18.48;22.64	<0.0001
Demand[2]			<0.0001			<0.0001
High	6.02	5.24;6.80		3.51	2.63;4.39	
Medium	2.93	2.34;3.52		1.76	1.08;2.44	
Low	0			0		
Time	-0.36	-0.46;-0.25	<0.0001			
Social support						<0.0001
No				2.66	1.57;3.76	
Yes				0		
Physical activity						<0.0001
Sedentary				4.44	3.59;5.30	
Light				1.43	0.86;2.00	
Moderate/intense				0		
SMBQlag				0.67	0.65;0.69	<0.0001
Age				-0.31	-0.06;-0.004	0.025
Intercept	68.38	67.85;68.91	<0.0001	20.72	18.65;22.78	<0.0001
Decision[2]			<0.0001			<0.0001
Low	3.58	2.98;4.17		1.99	1.38;2.60	
Medium	2.11	1.56;2.67		1.15	0.48;1.82	
High	0	0		0		
Time	-0.42	-0.53;-0.32	<0.0001			
Social support						<0.0001
No				2.71	1.61;3.80	
Yes				0		
Physical activity						<0.0001
Sedentary				4.36	3.51;5.22	
Light				1.30	0.73;1.87	
Moderate/intense				0		
SMBQlag				0.67	0.65;0.69	<0.0001
Age				-0.03	-0.05;0.001	0.060
Intercept	67.56	66.97;68.16	<0.0001	21.36	19.27;23.44	<0.0001
Effort[3]			<0.0001			<0.0001
High	5.67	5.00;6.35		3.61	2.85;4.36	

Medium	2.30	1.76;2.85		1.33	0.69;1.97	
Low	0			0		
Time	-0.30	-0.41;-0.20	<0.0001			
Social support						<0.0001
No				2.79	1.69;3.89	
Yes				0		
Physical activity						<0.0001
Sedentary				4.48	3.62;5.34	
Light				1.39	0.82;1.97	
Moderate/intense				0		
SMBQlag				0.66	0.64;0.68	<0.0001
Age				-0.04	-0.06;-0.008	0.011
Intercept	69.42	69.00;69.84	<0.0001	22.31	20.16;24.45	<0.0001
Reward[3]			<0.0001			<0.0001
Low	6.11	5.02;7.20		4.70	3.28;6.13	
Medium	3.18	2.51;3.86		2.71	1.83;3.60	
High	0	0		0		
Time	-0.38	-0.49;-0.26	<0.0001			
Social support						<0.0001
No				2.54	1.38;3.70	
Yes				0		
Physical activity						<0.0001
Sedentary				4.54	3.64;5.45	
Light				1.30	0.71;1.90	
Moderate/intense				0		
SMBQlag				0.66	0.63;0.68	<0.0001
Age				-0.03	-0.06;0.002	0.070
Intercept	69.42	68.99;69.84	<0.0001	21.30	19.25;23.35	<0.0001
JDC[4]			<0.0001			<0.0001
Joint exposure	5.37	4.55;6.19		3.67	2.69;4.65	
Demand only	3.12	2.36;3.88		1.56	0.58;2.53	
Decision only	2.03	1.49;2.56		1.20	0.62;1.78	
No exposure	0			0		
Time	-0.38	-0.49;-0.26	<0.0001			
Social support						<0.0001
No				2.64	1.54;3.74	
Yes				0		
Physical activity						<0.0001
Sedentary				4.39	3.54;5.25	
Light				1.39	0.81;1.96	
Moderate/intense				0		

SMBQlag				0.67	0.65;0.69	<0.0001
Age				-0.03	-0.06;-0.001	0.044
Intercept	68.83	68.39;69.27	<0.0001	21.07	19.42;22.71	<0.0001
ERI[5]			<0.0001			<0.0001
Joint exposure	7.87	6.32;9.41		5.83	3.79;7.85	
Effort only	3.93	3.37;4.50		2.84	2.16;3.52	
Reward Only	5.62	4.16;7.07		4.43	2.45;6.40	
No exposure	0			0		
Time	-0.32	-0.44;-0.21	<0.0001			
Social support						<0.0001
No				2.77	1.61;3.94	
Yes				0		
Physical activity						<0.0001
Sedentary				4.43	3.53;5.34	
Light				1.20	0.60;1.80	
Moderate/intense				0		
SMBQlag				0.65	0.63;0.68	<0.0001
Age				-0.03	-0.06;-0.003	0.032

[1]Shirom-Melamed Burnout Questionnaire, [2]Demand-Control Questionnaire, Decision Authority=subscale of the Control dimension, [3]Effort-Reward Questionnaire, [4] Job Demand-Control, [5]Effort-Reward Imbalance. *adjusted for time **Adjusted for social support, physical activity, age and SMBQlag.

Table 4 Longitudinal analysis showing lagged effects between burnout symptoms measured using SMBQ[1] and each of the workplace factors; regression coefficients (Coeff), 95% confidence interval (CI).

	*Model 1**			*Model 2***		
	Coeff.	*95% CI*	*p-value*	*Coeff.*	*95% CI*	*p-value*
Intercept	*68.12*	*67.30;68.95*	*<0.0001*	*21.69*	*19.86;23.51*	*<0.0001*
Demand[2]			*<0.0001*			*0.901*
High	2.84	1.94;3.74		0.18	-0.60;0.96	
Medium	1.07	0.40;1.75		0.11	-0.51;0.73	
Low	0			0		
Time	-0.13	-0.25;-0.01	0.033			
Social support						<0.0001
No				2.86	1.88;3.84	
Yes				0		
Physical activity						<0.0001
Sedentary				4.05	3.31;4.80	
Light				1.30	0.80;1.81	
Moderate/intense				0		
SMBQlag				0.68	0.66;0.70	<0.0001
Age				-0.04	-0.06;-0.01	0.002
Intercept	68.97	68.27;69.67	<0.0001	21.68	19.88;23.49	<0.0001
Decision[2]			0.059			0.679
Low	0.82	0.14;1.51		0.21	-0.34;0.75	
Medium	0.55	-0.10;1.10		0.01	-0.60;0.58	
High	0					
Time	-0.17	-0.29;-0.05	0.005			
Social support						<0.0001
No				2.78	1.80;3.76	
Yes				0		
Physical activity						<0.0001
Sedentary				4.02	3.27;4.76	
Light				1.30	0.79;1.80	
Moderate/intense				0		
SMBQlag				0.68	0.66;0.70	<0.0001
Age				-0.04	-0.06;-0.01	0.002
Intercept	68.52	67.72;69.32	<0.0001	21.91	20.07;23.75	<0.0001
Effort[3]			<0.0001			0.621
High	1.82	1.03;2.61		0.32	-0.37;1.00	
Medium	0.70	0.05;1.34		0.07	-0.52;0.66	
Low	0					
Time	-0.14	-0.27;-0.02	0.021			

Social support						<0.0001
No				2.91	1.92;3.90	
Yes				0		
Physical activity						<0.0001
Sedentary				3.97	3.22;4.73	
Light				1.26	0.75;1.77	
Moderate/intense				0		
SMBQlag				0.68	0.66;0.70	<0.0001
Age				-0.04	-0.07;-0.02	0.001
Intercept	68.79	68.18;69.41	<0.0001	22.79	20.92;24.67	<0.0001
Reward[3]			<0.0001			<0.0001
Low	3.54	2.28;4.80		1.18	0.02;2.34	
Medium	2.46	1.69;3.22		1.46	0.72;2.20	
High	0	0				
Time	-0.14	-0.27;-0.01	0.036			
Social support						<0.0001
No				2.74	1.71;3.77	
Yes				0		
Physical activity						<0.0001
Sedentary				4.20	3.42;5.00	
Light				1.41	0.89;1.94	
Moderate/intense				0		
SMBQlag				0.66	0.64;0.69	<0.0001
Age				-0.04	-0.06;-0.01	
Intercept	68.85	68.20;69.49	<0.0001	21.81	20.00;23.61	<0.0001
JDC[4]			<0.0001			0.198
Joint exposure	2.51	1.56;3.47		0.69	-0.16:1.54	
Demand only	1.57	0.70;2.44		-0.49	-1.31;0.33	
Decision only	0.41	-0.20;1.03		0.01	-0.51;0.53	
No exposure	0					
Time	-0.14	-0.27;-0.02	0.022			
Social support						<0.0001
No				2.80	1.82;3.79	
Yes				0		
Physical activity						<0.0001
Sedentary				4.02	3.27;4.77	
Light				1.29	0.79;1.80	
Moderate/intense				0		
SMBQlag				0.68	0.66;0.70	<0.0001
Age				-0.03	-0.06;-0.01	0.003
Intercept	68.80	68.15;69.44	<0.0001	22.71	20.80;24.61	<0.0001

ERI[5]			<0.0001			0.231
Joint exposure	3.88	2.08;5.69		1.36	-0.33;3.06	
Effort only	1.22	0.58;1.88		0.39	-0.20;0.98	
Reward Only	2.86	1.17;4.54		0.74	-0.83;2.32	
No exposure	0			0		
Time	-0.13	-0.26;0.01	0.054			
Social support						<0.0001
No				2.84	1.79;3.88	
Yes				0		
Physical activity						<0.0001
Sedentary				4.10	3.30;4.89	
Light				1.34	0.81;1.87	
Moderate/intense				0		
SMBQlag				0.67	0.65;0.69	<0.0001
Age				-0.04	-0.07;-0.01	0.003

[1]Shirom-Melamed Burnout Questionnaire, [2]Demand-Control Questionnaire, Decision Authority=subscale of the Control dimension, [3]Effort-Reward Questionnaire, [4] Job Demand-Control, [5]Effort-Reward Imbalance. *adjusted for time **Adjusted for social support, physical activity, age and SMBQlag.
